# Applications and potential of genome editing in crop improvement

**DOI:** 10.1186/s13059-018-1586-y

**Published:** 2018-11-30

**Authors:** Yi Zhang, Karen Massel, Ian D. Godwin, Caixia Gao

**Affiliations:** 1 Shandong Provincial Key Laboratory of Plant Stress, College of Life Science, Shandong Normal University, Jinan, 250014 China; 2 The University of Queensland, School of Agriculture and Food Sciences, St Lucia, QLD 4072 Australia; 3 The State Key Laboratory of Plant Cell and Chromosome Engineering, and Center for Genome Editing, Institute of Genetics and Developmental Biology, Chinese Academy of Sciences, Beijing, 100101 China; 4 University of Chinese Academy of Sciences, Beijing, 100049 China

## Abstract

Genome-editing tools provide advanced biotechnological techniques that enable the precise and efficient targeted modification of an organism’s genome. Genome-editing systems have been utilized in a wide variety of plant species to characterize gene functions and improve agricultural traits. We describe the current applications of genome editing in plants, focusing on its potential for crop improvement in terms of adaptation, resilience, and end-use. In addition, we review novel breakthroughs that are extending the potential of genome-edited crops and the possibilities of their commercialization. Future prospects for integrating this revolutionary technology with conventional and new-age crop breeding strategies are also discussed.

## Introduction

In today’s world, almost one billion people suffer from chronic malnourishment, while at the same time our agricultural systems are degrading, exacerbated by the loss of biodiversity and the increasing uncertainties of climate change [1]. With the global population projected to exceed 9 billion by 2050, contemporary agriculture will face enormous challenges, requiring crops with higher yields and of improved quality, and needing fewer inputs [2]. Although conventional breeding is currently the most widely used approach in crop improvement, it is labor intensive and it usually takes several years to progress from the early stages of screening phenotypes and genotypes to the first crosses into commercial varieties.

Genetically modified (GM) crops that have beneficial traits are produced by the transfer of genes (transgenes) or gene elements of known function into elite crop varieties. Despite the promise that GM crops hold for global food security, their use is affected by largely unsubstantiated health and environmental safety concerns. Government regulatory frameworks that aim to safeguard human and environmental biosafety have led to significant cost barriers to the rapid widespread adoption of new GM traits [3]. As a result, the advantages of GM traits have been restricted to a small number of cultivated crops.

**Fig. 1:**
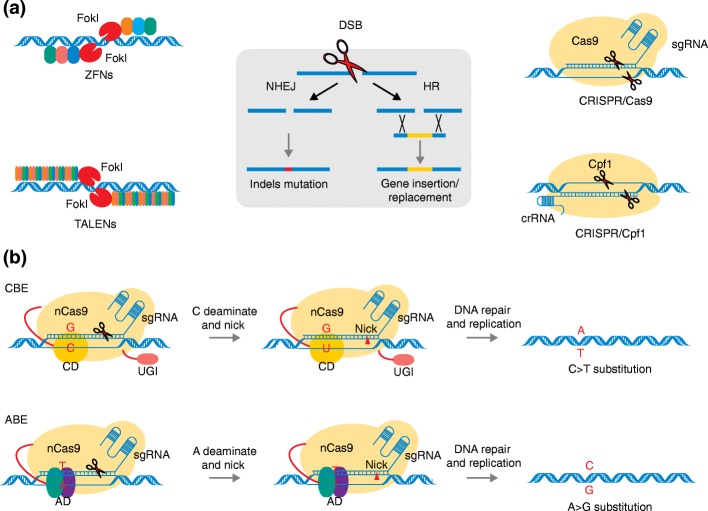
**a** Genome editing tools and DNA repair mechanisms. ZFNs and TALENs on the left panel use FokI endonuclease to cut DNA double strands. Since FokI functions as a dimer, when two ZFNs or TALENs bind their targets and bring the FokI monomers into close proximity, cleavage occurs. CRISPR/Cas9 system on the right panel employs sgRNA for DNA binding and Cas9 protein for DNA cleavage. While CRISPR/Cpf1 system uses crRNA for DNA binding and Cpf1 protein for DNA cleavage. On the middle panel, when DSB was produced by genome editing techniques, the plant’s endogenous repair systems fix the DSB by NHEJ or HR. NHEJ introduces small indels (red line) into the DSB and results in frame-shift mutations or premature stop codons. HR can cause gene replacements and insertions (yellow line) in the presence of a homologous donor DNA spanning the DSB. **b** Illustration of CRISPR/Cas9-mediated base editing. In the CBE system, nCas9 was fused to CD and UGI, and this complex could convert cytosine (C) in the targeting region to uracil (U), then U is changed to thymine (T) in DNA repair or replication processes, creating a C•G to T•A substitution. In the ABE system, nCas9 was fused to AD, and this system converts adenine (A) in the targeting region to inosine (I), which is treated as guanine (G) by polymerases, creating A•T to G•C substitutions. ABE adenine deaminases-mediated base editing, AD adenine deaminases, CBE cytidine deaminase-mediated base editing, CD cytidine deaminases, CRISPR clustered regularly interspaced short palindromic repeats, crRNA CRISPR RNA, DSB double-strand break, HR homologous recombination, nCas9 Cas9 nickase, NHEJ non-homologous end joining, sgRNA single-guide RNA, TALEN transcription activator-like effector nuclease, UGI uracil glycosylase inhibitor, ZFN zinc-finger nuclease

Genome editing is defined as a collection of advanced molecular biology techniques that facilitate precise, efficient, and targeted modifications at genomic loci [4, 5]. Genome editing using zinc-finger nucleases (ZFNs) [6] and transcription activator-like effector nucleases (TALENs) [7] has been around for two decades, but it has recently come under the spotlight through the development of clustered regularly interspaced short palindromic repeats (CRISPR)/Cas systems [8] which provide simplicity and ease of targeted gene editing (Fig. 1a). All of these technologies use typical sequence-specific nucleases (SSNs) that can be induced to recognize specific DNA sequences and to generate double-stranded breaks (DSBs) (Fig. 1a). The plant’s endogenous repair systems fix the DSBs either by non-homologous end joining (NHEJ), which can lead to the insertion or deletion of nucleotides thereby causing gene knockouts, or by homologous recombination (HR), which can cause gene replacements and insertions (Fig. 1a) [9]. Many gene knockout mutants and some gene replacement and insertion mutants have been produced through the use of genome-editing technologies in a wide variety of plants, and many of these mutants have been shown to be useful for crop improvement (Table 1).

**Table 1:** Crop traits that have been improved by genome-editing techniques

Crop species	Gene editor	Target gene	DNA repair type	Target trait	Reference
Maize	ZFNs	ZmIPK1	HR	Herbicide tolerant and phytate reduced maize	[14]
Maize	ZFNs	ZmTLP	HR	Trait stacking	[15]
Rice	ZFNs	OsQQR	HR	Trait stacking	[16]
Rice	TALENs	OsSWEET14	NHEJ	Bacterial blight resistance	[18]
Wheat	TALENs	TaMLO	NHEJ	Powdery mildew resistance	[19]
Maize	TALENs	ZmGL2	NHEJ	Reduced epicuticular wax in leaves	[20]
Sugarcane	TALENs	COMT	NHEJ	Improved cell wall composition	[21]
Sugarcane	TALENs	COMT	NHEJ	Improved saccharification efficiency	[22]
Soybean	TALENs	FAD2-1A, FAD2-1B	NHEJ	High oleic acid contents	[23]
Soybean	TALENs	FAD2-1A, FAD2-1B, FAD3A	NHEJ	High oleic, low linoleic contents	[24]
Potato	TALENs	VInv	NHEJ	Minimizing reducing sugars	[25]
Rice	TALENs	OsBADH2	NHEJ	Fragrant rice	[26]
Maize	TALENs	ZmMTL	NHEJ	Induction of haploid plants	[27]
*Brassica oleracea*	TALENs	FRIGIDA	NHEJ	Flowering earlier	[28]
Tomato	TALENs	ANT1	HR	Purple tomatoes with high anthocyanin	[29]
Rice	CRISPR/Cas9	LAZY1	NHEJ	Tiller-spreading	[39]
Rice	CRISPR/Cas9	Gn1a, GS3, DEP1	NHEJ	Enhanced grain number, larger grain size and dense erect panicles	[40]
Wheat	CRISPR/Cas9	GW2	NHEJ	Increased grain weight and protein content	[41]
*Camelina sativa*	CRISPR/Cas9	FAD2	NHEJ	Decreased polyunsaturated fatty acids	[42]
Rice	CRISPR/Cas9	SBEIIb	NHEJ	High amylose content	[43]
Maize	CRISPR/Cas9	Wx1	NHEJ	High amylopectin content	[44]
Potato	CRISPR/Cas9	Wx1	NHEJ	High amylopectin content	[45]
Wheat	CRISPR/Cas9	EDR1	NHEJ	Powdery mildew resistance	[46]
Rice	CRISPR/Cas9	OsERF922	NHEJ	Enhanced rice blast resistance	[47]
Rice	CRISPR/Cas9	OsSWEET13	NHEJ	Bacterial blight resistance	[48]
Tomato	CRISPR/Cas9	SlMLO1	NHEJ	Powdery mildew resistance	[49]
Tomato	CRISPR/Cas9	SlJAZ2	NHEJ	Bacterial speck resistance	[50]
Grapefruit	CRISPR/Cas9	CsLOB1 promoter	NHEJ	Alleviated citrus canker	[51]
Orange	CRISPR/Cas9	CsLOB1 promoter	NHEJ	Citrus canker resistance	[52]
Grapefruit	CRISPR/Cas9	CsLOB1	NHEJ	Citrus canker resistance	[53]
Cucumber	CRISPR/Cas9	eIF4E	NHEJ	Virus resistance	[54]
Mushroom	CRISPR/Cas9	PPO	NHEJ	Anti-browning phenotype	[55]
Tomato	CRISPR/Cas9	SP5G	NHEJ	Earlier harvest time	[56]
Tomato	CRISPR/Cas9	SlAGL6	NHEJ	Parthenocarpy	[57]
Maize	CRISPR/Cas9	TMS5	NHEJ	Thermosensitive male-sterile	[58]
Rice	CRISPR/Cas9	OsMATL	NHEJ	Induction of haploid plants	[59]
Tomato	CRISPR/Cas9	SP, SP5G, CLV3, WUS, GGP1	NHEJ	Tomato domestication	[60]
Rice	CRISPR/Cas9	ALS	HR	Herbicide resistance	[61]
Rice	CRISPR/Cas9	ALS	HR	Herbicide resistance	[62]
Rice	CRISPR/Cas9	EPSPS	NHEJ	Herbicide resistance	[63]
Rice	CRISPR/Cas9	ALS	HR	Herbicide resistance	[64]
Soybean	CRISPR/Cas9	ALS	HR	Herbicide resistance	[65]
Maize	CRISPR/Cas9	ALS	HR	Herbicide resistance	[66]
Potato	CRISPR/Cas9	ALS	HR	Herbicide resistance	[67]
Flax	CRISPR/Cas9	EPSPS	HR	Herbicide resistance	[68]
Cassava	CRISPR/Cas9	EPSPS	HR	Herbicide resistance	[69]
Maize	CRISPR/Cas9	ARGOS8	HR	Drought stress tolerance	[70]

*CRISPR* clustered regularly interspaced short palindromic repeats, *HR* homologous recombination, *NHEJ* non-homologous end joining, *TALEN* transcription activator-like effector nuclease, *ZFN* zinc-finger nuclease

The risks involved in altering genomes through the use of genome-editing technology are significantly lower than those associated with GM crops because most edits alter only a few nucleotides, producing changes that are not unlike those found throughout naturally occurring populations [10]. Once the genomic-editing agents have segregated out, there is no way to distinguish between a ‘naturally occurring’ mutation and a gene edit. Thus, the introduction of genome editing into modern breeding programs should facilitate rapid and precise crop improvement.

## Zinc-finger nucleases

ZFNs are fusions of zinc-finger-based DNA-recognition modules and the DNA-cleavage domain of the FokI restriction enzyme (Fig. 1a). Each individual zinc finger typically recognizes and binds to a nucleotide triplet, and fingers are often assembled into groups to bind to specific DNA sequences [11]. To date, ZFNs have been used to modify *Arabidopsis*, *Nicotiana*, maize, petunia, soybean, rapeseed, rice, apple, and fig (reviewed in [12, 13]). In one example of the application of ZFNs to crop breeding, the endogenous maize gene *ZmIPK1* was disrupted by insertion of *PAT* gene cassettes, and this resulted in herbicide tolerance and alteration of the inositol phosphate profile of developing maize seeds [14]. As a proven technology, ZFN-mediated targeted transgene integration was also used for trait stacking in maize, that is for assembling a number of useful traits together to create an even greater potential for crop improvement [15]. Later, Cantos et al. [16] used ZFNs to identify safe regions for gene integration in rice, and these identified sites should serve as reliable loci for further gene insertion and trait stacking. Nevertheless, the design of ZFNs remains a complicated and technically challenging process, and one that often has low efficacy.

## Transcription activator-like effector nucleases

Like ZFNs, TALENs are fusions of transcriptional activator-like effector (TALE) repeats and the FokI restriction enzyme (Fig. 1a) [17]. However, each individual TALE repeat targets a single nucleotide, allowing for more flexible target design and increasing the number of potential target sites relative to those that can be targeted by ZFNs. Genome editing by TALENs has been demonstrated in a wide variety of plants including *Arabidopsis*, *Nicotiana*, *Brachypodium*, barley, potato, tomato, sugarcane, flax, rapeseed, soybean, rice, maize, and wheat (reviewed in [12, 13]). The first application of TALEN-mediated genome editing in crop improvement was in rice, where the bacterial blight susceptibility gene *OsSWEET14* was disrupted and the resulting mutant rice were found to be resistant to bacterial blight [18]. Analogously, TALENs have been used in wheat to knockout three *TaMLO* homoeologs in order to create powdery mildew-resistant wheat [19]. By knocking out the maize *GL2* gene, Char et al. [20] obtained mutants with the glossy phenotype, with reduced epicuticular wax in the leaves and the potential to be surface manured. In sugarcane, cell wall composition and saccharification efficiency have been improved by TALEN-mediated mutagenesis [21, 22].

TALENs can be used to modify the nutritional profiles of crops: soybeans with high oleic acid and low linoleic acid contents were generated by disrupting fatty acid desaturase (*FAD*) genes, thus improving the shelf life and heat stability of soybean oil [23, 24]. In potato tubers, the accumulation of reducing sugars during cold storage influences the quality of the product, and knocking out the vacuolar invertase (*VInv*) gene resulted in tubers that had undetectable levels of problematic reducing sugars [25].

Flavor is very important, and fragrant rice has been produced through the use of TALEN technology to disrupt the betaine aldehyde dehydrogenase (*BADH2*) gene [26]. In addition, the production of haploid plants that inherit chromosomes from only one parent can greatly accelerate plant breeding. Using TALENs to create frame-shift mutations in *MATRILINEAL* (*MTL*) in maize, Kelliher et al. [27] obtained haploid maize. Also, by targeting *FRIGIDA* with TALENs, early-flowering mutants of *Brassica oleracea* were obtained [28].

Crop improvement by TALEN-mediated gene insertion is well exemplified in the tomato, where incorporating TALENs and donor DNA into geminivirus replicons significantly increased their copy number and hence the efficiency of homologous recombination [29]; a strong promoter was inserted upstream of the gene controlling anthocyanin biosynthesis, and purple tomatoes with high anthocyanin levels were obtained [29]. These examples demonstrate the vast potential of TALEN technology for crop trait improvement. However, the construction of TALE repeats remains a challenge and the efficiency of gene targeting with TALENs is variable.

## CRISPR/Cas9 system

CRISPR/Cas systems, especially the type II CRISPR/SpCas9 system from *Streptococcus pyogenes*, have been developed as versatile genome-editing tools for a wide variety of potential applications (Fig. 1a) [30]. Compared with ZFNs and TALENs, the CRISPR/Cas system is characterized by its simplicity, efficiency, and low cost, and by its ability to target multiple genes [31, 32]. Because of these characteristic features, CRISPR/Cas9 has been rapidly exploited in plants [33–35] and may be an effective solution to a variety of problems in plant breeding [36]. To date, many crops such as rice, maize, wheat, soybean, barley, sorghum, potato, tomato, flax, rapeseed, *Camelina*, cotton, cucumber, lettuce, grapes, grapefruit, apple, oranges, and watermelon have been edited by this technique (reviewed in [37, 38]). The most frequent application has been in the production of null alleles, or gene knockouts, predominantly achieved by the introduction of small indels that result in frame-shift mutations or by introducing premature stop codons (Fig. 1a).

Yield is a major concern in crop breeding. In rice, when the *LAZY1* gene was knocked out by CRISPR/Cas9, a tiller-spreading phenotype was generated, which could increase crop yield under certain circumstances [39]. Li et al. [40] used the CRISPR/Cas9 system to mutate the *Gn1a*, *DEP1*, and *GS3* genes of the rice cultivar Zhonghua11, producing mutants with enhanced grain number, dense erect panicles, and larger grain size, respectively. *Grain Weight 2* (*GW2*) is a key gene in cereal crops, which when disrupted increases grain weight and protein content in wheat [41].

The nutritional profiles of crops can also be improved by CRISPR/Cas9. As in the case of TALEN-mediated knockout in soybean to improve the shelf life and heat stability of soybean oil [23], CRISPR/Cas9 technology has been used to target *FAD2* to improve oleic acid content while decreasing polyunsaturated fatty acids in the emerging oil seed plant *Camelina sativa* [42]. In rice, Sun et al. [43] used CRISPR/Cas9 technology to generate targeted mutations in *SBEIIb*, leading to a higher proportion of long chains in amylopectin, which improved the fine structure and nutritional properties of the starch [43]. Using CRISPR/Cas9, DuPont Pioneer (now Corteva AgriScience) knocked out the maize waxy gene *Wx1*, which encodes the granule-bound starch synthase (GBSS) gene that is responsible for making amylose [44]. In the absence of GBSS expression in the endosperm, amylose was not synthesized, and this created a high amylopectin (waxy) maize with improved digestibility and the potential for bio-industrial applications [44]. The release of commercial hybrids with this trait is planned for 2020. The same gene has also been targeted in the potato by researchers at the Swedish Agricultural University to produce waxy potatoes, with improved cultivars aimed predominantly at the industrial starch market to be released in the next few years [45].

The technology also has been used to improve resistance to biotic stresses. Zhang et al. [46] used CRISPR/Cas9 technology to generate *Taedr1* wheat plants by simultaneous modification of the three homoeologs of *EDR1*. The resulting plants were resistant to powdery mildew and did not show mildew-induced cell death [46]. In rice, enhanced rice blast resistance and bacterial blight resistance were separately obtained by mutagenesis of OsERF922 and OsSWEET13 [47, 48]. Furthermore, powdery mildew-resistant tomatoes were generated by editing *SlMLO1* [49], and bacterial speck-resistant tomatoes were created by disrupting *SlJAZ2* [50]. Citrus canker is a severe disease that is responsible for significant economic losses worldwide, and *CsLOB1* is a susceptibility gene for citrus canker. By modifying the *CsLOB1* promoter, canker symptoms were alleviated in Duncan grapefruits [51] and Wanjincheng oranges had enhanced resistance to citrus canker [52]. CRISPR/Cas9 technology was later used to disrupt the coding region of *CsLOB1* in Duncan grapefruits, resulting in crops that had no canker symptoms [53]. In the cucumber, when the *eIF4E* (eukaryotic translation initiation factor 4E) gene was disrupted, broad virus resistance was generated [54]; the plants were shown to be immune to an Ipomovirus (Cucumber Vein Yellowing Virus) and were resistant to the potyviruses Zucchini yellow mosaic virus and Papaya ring spot mosaic virus-W [54].

Several other traits have been manipulated using CRISPR/Cas9 technologies. Polyphenol oxidase (PPO) is an enzyme that causes browning in many fruits and vegetables. By knocking out this gene, Waltz and coworkers [55] developed a non-browning mushroom. In the tomato, CRISPR/Cas9-engineered mutations in *SELF-PRUNING 5G* (*SP5G*) can result in rapid flowering [56], and a mutation in *SlAGAMOUS-LIKE 6* (*SlAGL6*) is responsible for the parthenocarpic phenotype [57]. In maize, when the thermosensitive genic male-sterile 5 gene (*TMS5*) was knocked out, thermosensitive male-sterile maize was generated [58]. Recently, haploid rice was induced by knockout of *OsMATL* by CRISPR/Cas9 [59]. Genome-editing techniques can also accelerate the domestication of crops. Using CRISPR/Cas9, Li et al. [60] introduced desirable traits into wild tomato accessions by targeting the coding sequences, cis-regulatory regions, and upstream open reading frames of genes associated with tomato morphology, flower and fruit production, and ascorbic acid synthesis [60].

CRISPR/Cas9-mediated editing by gene insertion and replacement has been used to create herbicide-resistant crops. Herbicide-resistant rice has been developed by a variety of methods such as disrupting DNA ligase 4, which is implicated in NHEJ repair [61], using two single-guide RNAs (sgRNAs) targeting the repair template [62], NHEJ-mediated intron targeting [63], and the use of chimeric single-guide RNAs (cgRNAs) carrying both target site and repair template sequences [64]. When the targeting efficiency was high enough, herbicide-resistant soybean and maize could be created by co-transforming CRISPR/Cas9 and donor DNAs by particle bombardment [65, 66]. Using geminivirus replicons that increase the copy number of CRISPR/Cas9 and a repair template, Butler et al. [67] produced herbicide-resistant potatoes. Moreover, herbicide-resistant flax has been generated using a combination of single-stranded oligonucleotides and CRISPR/Cas9 [68]. Recently, a promoter swap and dual amino-acid substitutions were achieved at the *EPSPS* locus in cassava, generating glyphosate tolerance [69]. In addition to producing herbicide-resistant crops, CRISPR/Cas9-mediated gene insertion and replacement methods have created drought-resistant properties in maize [70]. The GOS2 promoter confers a moderate level of constitutive expression, and when it was inserted into the 5′-untranslated region of the native *ARGOS8* gene, or when it replaced the endogenous ARGOS8 promoter, increased *ARGOS8* transcripts were detected and resulted in increased drought tolerance [70].

## Novel technical breakthroughs

Genome-editing technology already shows great potential in agriculture, but it is still limited by the low efficiency of HR, off-target effects, restrictive protospacer adjacent motif (PAM) sequences, and other issues. Fortunately, novel innovations are continually being added to the genome-editing toolkit to address these limitations.

### Base editing

To date, HR repair of DSBs using template donor DNA has been found to be much less efficient than template-free NHEJ, making it difficult to induce single nucleotide substitutions (rather than indels) in plants. However, genome-wide association studies have shown that single-base changes are usually responsible for variations in elite traits in crop plants [71]; hence, efficient techniques for producing precise point mutations in crops are needed urgently. CRISPR/Cas9-mediated base-editing technology is a new genome-editing approach that can accurately convert one DNA base into another, without the use of a DNA repair template [72]. The base-editing technologies employ Cas9 nickase (nCas9) or dead Cas9 (dCas9) fused to an enzyme with base conversion activity. For example, cytidine deaminases convert cytosine (C) to uracil (U), and the latter is treated as thymine (T) in subsequent DNA repair or replication processes, so creating a C•G to T•A substitution (Fig. 1b) [72, 73]. Likewise, adenine deaminases convert adenine (A) to inosine (I), which is treated as guanine (G) by polymerases, creating A•T to G•C substitutions (Fig. 1b) [74]. Cytidine-deaminase-mediated base editing (CBE) has been used in rice, *Arabidopsis*, wheat, maize, and tomato (reviewed in [75, 76]). Recently, this technology has been used in watermelon and wheat to create herbicide-resistant plants [77, 78].

Adenine-deaminase-mediated base editing (ABE) is more complicated than CBE because no known naturally occurring cytidine deaminases catalyze adenine deamination in DNA rather than RNA. Fortunately, Gaudelli and colleagues [74], using several rounds of directed evolution and protein engineering, were able to develop an efficient ABE. In rice, Yan et al. [79] generated a fluorescence-tracking A to G base editor. Hua et al. [80] also developed an adenine base editor in rice and used it in multiplex base editing. An ABE has also been used with rapeseed protoplasts and in *Arabidopsis*, and the desired phenotypic alterations and germline transmission were observed in *Arabidopsis* [81]. Li et al. [82] improved the ABE system for producing edited rice and wheat plants, and generated point mutations within the acetyl-coenzyme A carboxylase (*ACC*) gene in rice that conferred herbicide-resistance.

In addition to generating point mutations, CBE can also be used to produce nonsense mutations that disrupt genes of interest and knockout their gene functions [83]. CBE is much more specific than conventional SSN-mediated knockout, causing few if any indels. All-in-all, base-editing tools have given genome editing a new dimension, broadening its potential applications by means of nucleotide-specific modifications at specific genomic sites.

### DNA-free genome editing systems

Conventional genome editing involves the delivery and integration into the host genome of DNA cassettes encoding editing components. Integration occurs at random, and therefore can generate undesirable genetic changes. Even if the DNA cassettes are degraded, the resulting fragments may be integrated and could produce undesirable effects [84]. Prolonged expression of genome-editing tools increases off-target effects in plants since nucleases are abundant in these organisms [19, 26, 85]. Moreover, the introduction of foreign DNA into plant genomes raises regulatory concerns in relation to GM organisms [86]. Therefore, DNA-free genome editing is a groundbreaking technology, producing genetically edited crops with a reduced risk of undesirable off-target mutations, and meeting current and future agriculture demands from both a scientific and regulatory standpoint.

DNA-free genome editing has been accomplished using both protoplast-mediated transformation and particle bombardment. The first successful report of DNA-free genome editing in plants was by Woo and colleagues [87] who transfected CRISPR/Cas9 ribonucleoproteins (RNPs) into protoplasts of *Arabidopsis*, tobacco, lettuce, and rice. Similarly, Malnoy et al. [88] produced targeted mutations by delivering purified CRISPR/Cas9 RNPs into protoplasts of both grape and apple. Unfortunately, efficient, regenerable protoplast systems are not available for a number of agriculturally important higher crop species, and therefore there has been a search for other DNA-free genome editing methods.

Particle bombardment-mediated DNA-free genome-editing technology has been developed in wheat and maize [89–91]. Both CRISPR/Cas9 RNA and CRISPR/Cas9 RNPs have been delivered into wheat embryos by particle bombardment, and both methods created genome-edited plants [89, 90]. In maize, CRISPR/Cas9 RNPs have been used not only to generate knockout mutants, but also to obtain targeted knockin mutants with the help of single-stranded DNA oligonucleotides [91]. Unlike CRISPR/Cas9 editing with DNA cassettes, CRISPR/Cas9 RNPs cause few if any off-target effects in plants and have a relatively high editing efficiency [90, 91].

Recently, a combination of base editing and DNA-free genome editing has been described in wheat [78], with an average frequency of C-to-T conversion of 1.8%. This development should greatly facilitate both the application of base editing to plant breeding and the commercialization of edited plants.

### CRISPR/Cpf1 system

The type II CRISPR/SpCas9 system is simple and efficient, but it can only recognize DNA sequences upstream of the appropriate 5’-NGG-3’ PAMs, thus restricting potential target sites. Therefore, Cas9 variants were needed to overcome this limitation. The type V CRISPR/Cpf1 system has demonstrated great potential in this area. Cpf1 recognizes T-rich PAMs and generates cohesive ends with four or five nucleotide overhangs rather than blunt-end breaks, which complements the characteristics of Cas9 to a large extent (Fig. 1a) [92]. Recently, Cpf1 from *Francisella novicida* (FnCpf1) was used for targeted mutagenesis in tobacco and rice [93], and the Cpf1 ortholog from a Lachnospiraceae bacterium (LbCpf1) generated targeted mutations in rice [94, 95]. A variant AsCpf1 (Cpf1 ortholog from *Acidaminococcus* sp. BV3L6) demonstrated high genome-editing efficiencies in human cells [96], but was less efficient in rice [97] and in soybean and rice protoplasts [98, 99].

When tested for their ability to induce targeted gene insertions via HR, the FnCpf1 and LbCpf1 nucleases generated precise gene insertions at a target site in rice, at a higher frequency than most other genome-editing nucleases [100]. LbCpf1 has also been used for targeted gene replacement in rice [101]. Recently, to expand the scope of CRISPR/Cpf1-mediated genome editing in rice, Li et al. [102] developed an LbCpf1 (RR) variant that enables the editing and multiplex editing of target genes containing TYCV PAMs.

Like the CRISPR/Cas9 system, the CRISPR/Cpf1 system may be combined with base editing and/or DNA-free genome editing. In fact, CRISPR/Cpf1-mediated DNA-free genome editing has been achieved in rice [98]. As CRISPR/Cpf1-mediated base editing using a T-rich PAM sequence has produced C-to-T conversions in human cells [103], similar applications in crop plants should not be too far in the future.

## Prospects and future directions

### Multiplexing and trait stacking in crop breeding

In plants, cellular processes are often regulated by complex genetic networks, and the manipulation of agronomic traits depends on the precise engineering of complex metabolic pathways, which requires the concerted expression of multiple genes. Therefore, molecular tools with the capability to manipulate multiple genes simultaneously are of great value in both basic research and practical applications.

One of the advantages of CRISPR systems over other genome-editing methods is their potential for multiplexing, the simultaneous editing of multiple target sites [31]. Using Golden Gate cloning or the Gibson Assembly method, several groups have assembled multiple sgRNAs into single Cas9/sgRNA expression vectors, in which multiple sgRNAs are driven by separate promoters (reviewed in [104]). Xie et al. [105] have developed a general strategy for producing numerous sgRNAs from a single polycistronic gene. They engineered the endogenous tRNA-processing system, creating a simple and robust platform for expanding the targeting and multiplex editing capability of the CRISPR/Cas9 system. This tRNA-processing system has also been employed for multiplex editing in the CRISPR/Cpf1 system [106]. Cpf1 differs from Cas9 in being a dual nuclease that not only cleaves target DNA but also processes its own CRISPR RNA [107, 108]. Taking advantage of this characteristic, Wang et al. [109] engineered CRISPR/Cpf1 together with a short DR-guide array in rice and demonstrated the feasibility of multiplex gene editing. Multiple sgRNAs can also be used to target a single gene to improve rates of editing in crops that have low transformation or editing efficiencies.

### High-throughput mutant libraries

Now that the complete genomes of many crops have been sequenced, the challenge of the post-genomic era is to analyze the functions of all crop genes systematically, as most of the genes sequenced to date have unknown functions and may control important agronomic traits. Gene knockout is a frequently used and effective strategy for identifying gene functions; hence, large-scale mutant libraries at the whole-genome level are of great value for functional genomics and for crop improvement.

Genome-wide mutant libraries in rice have been constructed by two teams. Lu et al. [110] designed 88,541 sgRNAs targeting 34,234 genes to create a total of 91,004 targeted loss-of-function mutants. Meng et al. [111] designed 25,604 sgRNAs corresponding to 12,802 genes and obtained more than 14,000 transgenic T0 lines. These two groups selected rice for genome-wide targeted mutagenesis mainly because of its relatively small genome, rich genomic resources, and highly efficient transformation system. As techniques evolve, the construction of mutant libraries in other valued crop species should not be too long delayed.

### Gene regulation

Besides gene knockouts and knockins, genome editing tools can also be used to regulate gene expression. Gene regulation mainly involves the repression and activation of genes and is often achieved by fusing transcriptional repressors or activators to the DNA-binding domains of genome-editing constructs (such as zinc finger protein (ZFP), TALE, or dCas9), thereby targeting the regulatory regions of endogenous genes [112]. In rapeseed, the VP16 transcriptional activation domain was fused to ZFP, which binds to the DNA sequence downstream of the transcription start site of *KASII* genes. Mutants in which *KASII* was activated displayed the desirable agronomic trait of decreased levels of palmitic acid and total saturated fatty acid [113]. CRISPR/Cas9 can also be used to repress or activate the transcription of plant genes by combining catalytically inactive dCas9 with sgRNAs that target specific promoter sequences [114, 115]. Furthermore, both AsCpf1 and LbCpf1 have been used to repress transcription in *Arabidopsis,* thus underlining the great promise of Cpf1 for modulating plant transcriptomes [99].

Recently, CRISPR/Cas9 technology has been used for crop improvement by altering the cis-regulatory control of quantitative trait loci. Rodriguez-Leal et al. [116] used CRISPR/Cas9 to mutate the SlCLV3 promoters in tomato and produced hundreds of regulatory mutations. In this way, they could systematically assess the association of cis-regulatory regions with phenotypic traits, which should be helpful in enhancing tomato breeding. Zhang et al. [117] reported that endogenous plant upstream open reading frames (uORFs) could be edited by CRISPR/Cas9 technology to modulate the translation of mRNAs. Targeting the uORF of *LsGGP2* generated a mutant lettuce with improved tolerance to oxidative stress and increased ascorbate content [117]. This strategy provides a generalizable, efficient method for manipulating the translation of mRNAs, which can be applied to dissect biological mechanisms and improve crops.

Unlike applications that aim primarily to alter DNA sequences, the effects of genome editing on gene regulation act at the transcript level, and could be used to reveal the function of many non-canonical RNAs that are related to crop improvement. As most non-coding transcripts are nuclear and lack open reading frames, genome editing that modulates transcription directly is optimally suited to interrogating the function of such RNAs.

## Conclusions

Over the past several decades, traditional breeding that depends on access to plant populations with sufficient variability has made great contributions to agriculture. However, this variability is mainly derived from spontaneous mutations or from mutations that are induced by chemical mutagens or physical irradiation. Such mutations are usually rare and occur at random. Moreover, many types of variation might not arise in elite varieties, and consequently time-consuming, laborious breeding programs are needed to introduce desirable alleles into elite crops. By contrast, genome editing as an advanced molecular biology technique can produce precisely targeted modifications in any crop [4, 5].

**Fig. 2:**
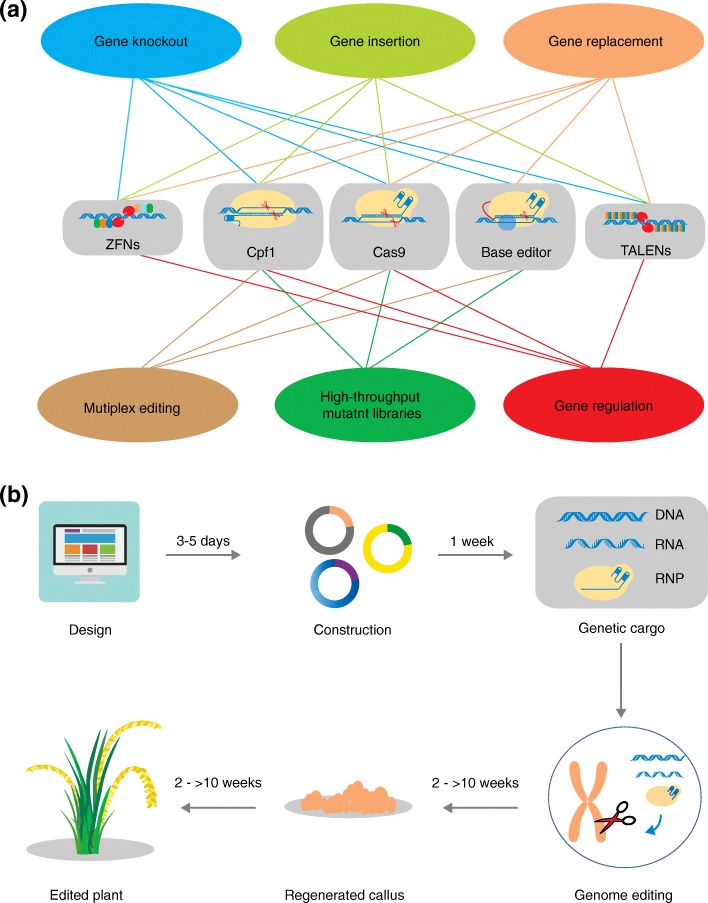
**a** The network of genome editing methods and the corresponding genome editing tools. **b** Flow chart illustrating the successive steps in plant genome editing, and the estimated time needed for each step. RNP ribonucleoprotein, TALEN transcription activator-like effector nuclease, ZFN zinc-finger nuclease

In this review, we have described the current applications of three standard genome-editing techniques for crop improvement, and have introduced the relatively new base-editing and CRISPR/Cpf1 systems, which also have great potential in agriculture. Given the availability of a variety of genome-editing tools with different applications (Fig. 2a), it is important to consider the optimal system for a given species and purpose. Once appropriate genome-editing tools have been selected, the target sequences are designed and introduced into the most suitable vectors, and the appropriate genetic cargo (DNA, RNA, or RNPs) for delivery is selected (Fig. 2b). After the genetic cargo has entered the target plant cells, the target sequences will be modified, and edited calli will be regenerated and will ultimately give rise to edited plants (Fig. 2b).

It may well be that protoplast-based systems are not readily available, or even possible, in a species of choice. Furthermore, regeneration by tissue culture may be difficult or limited to a few model genotypes. In these cases, it may be beneficial to design methodologies that do not require regeneration, such as the use of pollen, or the use of immature embryos that can be coaxed to germinate in vitro. With the progress already made in the development of genome-editing tools and the development of new breakthroughs, genome editing promises to play a key role in speeding up crop breeding and in meeting the ever-increasing global demand for food. Moreover, the exigencies of climate change call for great flexibility and innovation in crop resilience and production systems. In addition, we must take into account government regulations and consumer acceptance around the use of these new breeding technologies.

## Abbreviations

ABE: Adenine deaminase-mediated base editing
AsCpf1: Cpf1 ortholog from *Acidaminococcus* sp. BV3L6
CBE: Cytidine deaminase-mediated base editing
CRISPR: Clustered regularly interspaced short palindromic repeats
dCas9: Dead Cas9
DSB: Double-stranded break
*FAD*: fatty acid desaturase
FnCpf1: Cpf1 from *Francisella novicida*
GBSS: granule-bound starch synthase
GM: Genetically modified
HR: Homologous recombination
LbCpf1: Cpf1 from Lachnospiraceae bacterium
NHEJ: Non-homologous end joining
PAM: Protospacer adjacent motif
RNP: Ribonucleoprotein
sgRNA: Single-guide RNA
SSN: Sequence-specific nuclease
TALE: Transcriptional activator-like effector
TALEN: Transcription activator-like effector nuclease
ZFN: Zinc-finger nuclease
ZFP: Zinc finger protein
