# Correction to: Applications and potential of genome editing in crop improvement

**DOI:** 10.1186/s13059-019-1622-6

**Published:** 2019-01-16

**Authors:** Yi Zhang, Karen Massel, Ian D. Godwin, Caixia Gao

**Affiliations:** 1 Shandong Provincial Key Laboratory of Plant Stress, College of Life Science, Shandong Normal University, Jinan, 250014 China; 2 The University of Queensland, School of Agriculture and Food Sciences, QLD, St Lucia, 4072 Australia; 3 The State Key Laboratory of Plant Cell and Chromosome Engineering, and Center for Genome Editing, Institute of Genetics and Developmental Biology, Chinese Academy of Sciences, Beijing, 100101 China; 4 University of Chinese Academy of Sciences, Beijing, 100049 China

**Correction to: Genome Biology**

**https://doi.org/10.1186/s13059-018-1586-y**

Following publication of the original article [1], the authors reported the following two errors:

1. Page 5, paragraph 3, line 4: “Polyphenol oxidase (PPO) is an enzyme that causes browning in many fruits and vegetables. By knocking out this gene, Waltz and coworkers developed a non-browning mushroom”. “Waltz and coworkers” should be “Yinong Yang and coworkers”.
2. Page 6, paragraph 2, line 3: “Adenine-deaminase-mediated base editing (ABE) is more complicated than CBE because no known naturally occurring cytidine deaminases catalyze adenine deamination in DNA rather than RNA”. “naturally occurring cytidine deaminases” should be “naturally occurring adenine deaminases”.
